# Area-level socioeconomic deprivation and mortality differentials in Thailand: results from principal component analysis and cluster analysis

**DOI:** 10.1186/s12939-017-0613-z

**Published:** 2017-07-03

**Authors:** Suchunya Aungkulanon, Viroj Tangcharoensathien, Kenji Shibuya, Kanitta Bundhamcharoen, Virasakdi Chongsuvivatwong

**Affiliations:** 1 Epidemiology Unit, Faculty of Medicine, Prince of Songkla University, Songkhla, Thailand; 2 International Health Policy Program, Ministry of Public Health, Nonthaburi, Thailand; 3 Department of Global Health Policy, Graduate School of Medicine, University of Tokyo, Tokyo, Japan

**Keywords:** Socioeconomic status, Mortality, Health inequality, Thailand

## Abstract

**Background:**

Despite achievement of universal health coverage in Thailand, socioeconomic inequality in health has been a major policy concern. This study examined mortality patterns across different socioeconomic strata in Thailand.

**Methods:**

We conducted a cross-sectional analysis of the 2010 Population and Housing Census on area-level socioeconomic deprivation against the 2010 mortality from the vital registration database at the super-district level. We used principal components analysis to construct a socioeconomic deprivation index and K-mean cluster analysis to group socioeconomic status and cause-specific mortality.

**Results:**

Excess mortality rates from all diseases, except colorectal cancer, were observed among super-districts with low socioeconomic status. Spatial clustering was evident in the distribution of socioeconomic status and mortality rates. Cluster analysis revealed that super-districts which were predominantly urban tended to have low all-cause standardize mortality ratio but a high colorectal cancer-specific mortality rate. Deaths due to liver cancer, diabetes, and renal diseases were common in the low socioeconomic super-districts which hosted one third of the total Thai population.

**Conclusion:**

Socially deprived areas have an excess of overall and cause specific deaths. Populations living in more affluent areas, despite low general mortality, still have many preventable deaths such as colorectal cancer. These findings warrant future epidemiological studies investigating various causes of excessive deaths in non-deprived areas and implementation of policies to reduce the mortality gap between rich and poor areas.

**Electronic supplementary material:**

The online version of this article (doi:10.1186/s12939-017-0613-z) contains supplementary material, which is available to authorized users.

## Background

Socioeconomic inequality among different regions has been rising in Thailand and in other countries [1, 2]. Apart from socioeconomic disparities, geographical variations in all causes and cause-specific mortality rates also exist in Thailand [3]. Previous studies revealed high mortality rates in the northern region but high mortality rates due to liver cancer and diabetes in the northeastern region [4]. Bangkok metropolitan area is known to have a low overall mortality rate compared to other regions, but a high rate due to cardiovascular diseases.

Socioeconomic status at the geographical levels has been identified as a major determinant of health in a population [5]. The socioeconomic characteristics of an area influences the health of a population through various mechanisms such as physical characteristics, for example availability of goods and services and environmental pollutants, and social characteristics, for example societal cohesion, collective efficacy (reflecting the ability of members of a community to control the behavior of individuals or groups in the community ensuring safety and security) and social support to cope with stress [6].

Socioeconomically disadvantaged areas contribute to higher mortality than the more advantaged areas [7–10]. To date, most of the evidence on neighborhood socioeconomic inequalities in health has been reported from high income countries. This issue remains largely unexplored in low and middle income countries.

Studies of investigating geographical and socioeconomic determinants of health have often applied an area-level deprivation index which is constructed by combining several parameters such as education, income, and various other demographic characteristics [5]. Principal component analysis (PCA) is commonly used to construct deprivation index. This deprivation index can be then used to correlate with health outcomes such as mortality rates.

However, the use of PCA sometimes generates ambiguity due to its over-simplifications [11, 12]. The use of such aggregate measures cannot identify the distinct characteristics attributable to specific geographical areas that can often explain the variations in cause-specific mortality rates. A more complete understanding of mortality patterns by geographical and socioeconomic characteristics should contribute to better planning and equitable resource allocation for different areas.

Cluster analysis is another statistical method which can be used to improve our understanding of the health and socioeconomic situation of a population by summarizing the main patterns across a wide range of variables. Using this approach, the main patterns can be summarized into a series of groups or areas, which can subsequently be classified by their mortality pattern and socioeconomic type [13].

Choropleth maps can be used to display the geographical distribution of measurements of a population such as mortality rate and socioeconomic status. Together with cross-tabulation of mortality rate and socioeconomic status, the map can allow a visualization of the association between the two variables on a spatial dimension, which can then provide evidence for policy makers to invest more in health services to the most disadvantaged regions as well as specific health interventions to reduce cause specific mortality. Such a map will facilitate better targeting of health interventions and resource investments [14]. This study examined the deprivation and cause-specific mortality, mortality clustering, socioeconomic clustering and correlation between mortality clusters and socioeconomic clusters in Thailand.

## Methods

**Fig. 1:**
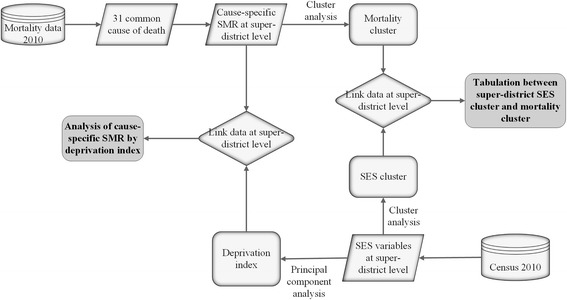
Methodology flowchart

A cross-sectional analysis of two datasets was conducted; the 2010 Census of Population and Housing conducted by the National Statistical Office and mortality records from the Civil Registration and Vital Statistics in 2010. A flowchart of the methodology is shown in Fig. 1, which describes the process of analysis of these two datasets. From the flow chart, four main findings emerge: cause specific standardized mortality ratio (SMR) by deprivation index at the super-district level, mortality clusters, geographical socioeconomic clusters, and a tabulation between geographical socioeconomic and mortality clusters.

### Unit of analysis

In 2010, Thailand was classified as a lower middle-income country with a population of 63 million. There were 77 provinces and 928 administrative districts. The population of these districts varied substantially, ranging from a minimum of 2253 to a maximum of 492,490. The significant variation in population size of the districts violates the assumption of equal variance within each unit. To alleviate this violation, we aggregated adjacent districts, thus systematically created what we call super-districts. These super-districts have less variation in population size, thus the mortality rates in areas in these are relatively more stable. A detailed explanation for deriving super-districts is described elsewhere [4]. The 928 administrative districts were aggregated into 331 super-districts, with a median population of 189,067 persons and ranging from 100,970 to 492,490. These super-district units were used throughout the analysis.

### Data sources

#### Mortality data

Population mortality data in 2010 were obtained from the national vital registration system, responsible by the Ministry of Interior, Bureau of Registration Administration. Each record includes date of birth and date, cause and place of death. The description of the cause of death was coded by the Bureau of Policy and Strategy, Ministry of Public Health using the International Classification of Diseases, 10th Revision (ICD-10). Individual mortality was credited to each super-district according to registered place of residence of the deceased. The leading cause of death was categorized by the condensed ICD-10 mortality tabulation list 1, which contains 103 causes of death [15]. We selected the 31 most common causes of death based on mortality in the general population, which covered 95% of all deaths in 2010.

Standardized mortality ratios were calculated to compare mortality across super-districts. The numbers of deaths expected in each super-district was calculated by taking the reference from the mortality rates of Thailand for the year 2010, by sex, age and cause of death. Mid-year population data in 2010 was obtained from the Bureau of Registration Administration, Ministry of Interior. The SMR was the main measure of mortality in all subsequent analyses.

#### Socioeconomic data

A random weighted sample of 20% of the 2010 Census of Population and Housing database was obtained from the National Statistics Office, which was the maximum proportion that the research team was allowed to access. This proportion was deemed adequate for the analysis at the super-district level. The census includes the following variables at the household and individual levels: demographic characteristics such as age, gender, nationality, marital status, education level and literacy, occupation and work status, migration characteristics and duration of living in the house, characteristic and tenure of dwelling, and ownership of durable appliances [16]. Income and expenditure information are not included in the Thai census.

Based on previous studies on important socioeconomic indicators which contribute to the construction of the deprivation index [17–28] and availability of the data, we identified 18 socio-demographic variables in the census, listed in Table 1, to construct the deprivation index. Similar to the mortality data, socioeconomic data of individuals and households were aggregated to obtain the proportion of individuals and households at the super-district level. Each aggregated variable was standardized into a z-score and used in the principle component analysis and cluster analysis.

**Table 1:** List of socioeconomic and demographic variables in the census to construct the deprivation index

Domain	Variable	Description	Rationale for chosen variable
Location	Rural residency	% of households in non-municipal areas	Literature review [20]
Demography	Unattached elderly	% of elderly persons, (aged 60 and over) living alone	Literature review [17]
	Divorced/separated/widowed	% of females aged 15 and over who are separated, divorced or widowed	Literature review [27]
	Age dependency ratio	Ratio of the population aged under 15 or over 60 to the the population aged between 15 and 60	Available census data
	Non-Thai	% of non-Thai citizens	Available census data
Education	Low education	% of persons aged over 15 years with less than primary or primary education	Literature review [23]
	Illiterate	% of persons aged over 15 years who are illiterate	Literature review [25]
Disability	Disabled	% of persons with visible disabilities	Available census data
Employment	Unemployment	% of persons aged over 15 years who are unemployed	Literature review [19, 23, 26, 27]
Housing	No dwelling ownership	% of not owning their dwelling	Literature review [19, 21, 26, 28]
	No vehicle ownership	% of households without car/motorbike	Literature review [19, 24, 28]
	No refrigerator ownership	% of households with no refrigerator	Available census data
	No television ownership	% of households with no television	Available census data
	No phone ownership	% of households without access to phone	Available census data
	No internet access	% of households without access to the Internet	Available census data
Crowdedness	Overcrowded home	% of households with more than 1 person per bedroom	Literature review and available census data [24]
	Children 3+	% of couples who are married with 3 or more children	[22]
Residential mobility	Migrant	% of persons with a different address 1 year before the census	Literature review and available census data [18, 24, 26]

### Statistical analysis

#### Principle component analysis

Principal component analysis (PCA) is a data reduction technique frequently used to create socio-demographic scales or indices [29, 30]. In constructing the deprivation index, 18 socioeconomic variables were reduced into a single factor using varimax rotation. Factor scores from the first principal component was designated as the deprivation index. Higher scores correspond to higher levels of deprivation. Super-districts were then classified into quintiles according to their deprivation index. A choropleth map was created to visualize the geographical distribution of socioeconomic deprivation by quintile. PROC FACTOR procedures in SAS version 9.4 was used to conduct the PCA.

#### Analysis of socioeconomic gradient and cause-specific mortality

To examine the effect of simplified socioeconomic gradient on mortality, the cause-specific SMR between the two extremes - the top quintile (Q5: most deprived) and the bottom quintile (Q1: least deprived) - were compared to reflect the effect of socioeconomic gradient on cause-specific mortality. Note that this is unlike wealth index quintiles where Q1 represents the poorest group and Q5 represents the richest.

#### Cluster analysis

This study adopted the K-means clustering method to separately group super-districts based on socioeconomic status and mortality rate. K-means clustering is a well-known and widely used technique due to its simplicity, robustness, and efficiency when using large datasets [31]. It classifies observations into clusters in which each observation belongs to the cluster with the nearest mean.

The procedure begins with the construction of initial cluster centers. Each super-district is individually relocated to the cluster center that they are located nearest to and then the relocation is assessed to see if it improves the model. Observations are then reassigned until no further improvements can be gained. The cluster algorithm is repeated for all values of *k* in the range 1 to 9, where k represents the number of clusters. The appropriate number of clusters was chosen based the following criteria: 1) generating interpretable clustering patterns; 2) having neither too few nor too many small clusters; and 3) assessing an elbow plot of the overall R^2^ which can help to determine when a further increase in the number of clusters would result in a relatively little increase in R^2^. PROC FASTCLUS procedures in SAS version 9.4 was used to conduct the cluster analysis. A choropleth map of Thailand was created to visualize the geographical patterns of mortality and socioeconomic status across all super-districts of Thailand.

#### Association between socioeconomic status and mortality

Fisher’s exact test was used to assess the association between socioeconomic patterns and mortality clusters.

## Results

### Deprivation index and cause-specific mortality

**Fig. 2:**
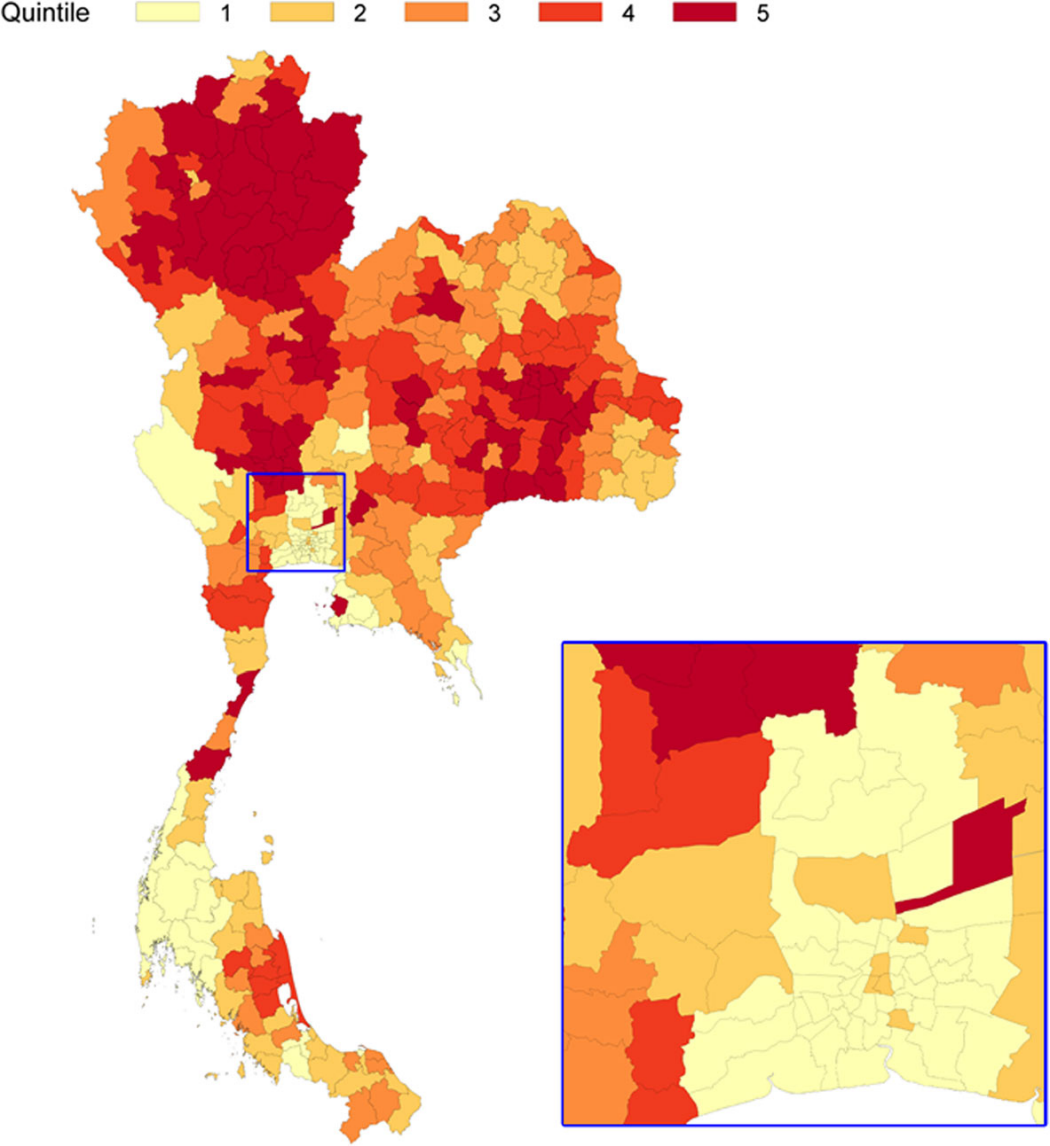
Geographical distribution of deprivation index

The first principal component explained 40.8% of the total variation in socioeconomic status. This was designate as the deprivation index. A map of super-districts showing variation in the social deprivation gradient is displayed in Fig. 2 with darker hue indicating higher social deprivation. The most deprived areas were mainly located in the northern region and some parts of the northeastern and central regions. Greater Bangkok was the least deprived area.

Table 2 compares cause-specific SMR of Q1 (least deprived super-districts) and Q5 (most deprived super-districts) by the top 31 causes of mortality. The last column (the extreme ratio between Q5 and Q1) indicates the level of inequality in cause specific mortality between the most and least deprived super-districts.

**Table 2:** Cause-specific mortality by deprivation quintiles

Rank	Cause of death	Number of deaths	Cause-specific SMR					Ratio Q5 to Q1 (95% CI)
			Quintile 1 (least deprived)	Quintile 2	Quintile 3	Quintile 4	Quintile 5 (Most deprived)	
1	Unspecified	154,685	0.71	0.93	1.07	1.06	1.12	1.59 (1.47–1.72)
2	Sepsis	28,959	1.10	0.87	0.86	0.81	0.95	0.86 (0.76–0.97)
3	Cerebrovascular diseases	17,538	1.01	0.93	0.83	0.82	1.04	1.03 (0.92–1.14)
4	Liver and bile duct cancer	16,850	0.55	0.66	0.86	1.01	1.03	1.87 (1.49–2.33)
5	Pneumonia	14,886	1.11	0.88	0.76	0.81	0.89	0.81 (0.69–0.94)
6	Traffic injury	13,772	0.50	1.03	1.07	0.99	1.05	2.08 (1.77–2.45)
7	Other external cause	13,668	0.94	0.86	0.89	0.98	1.03	1.10 (0.98–1.24)
8	Other cancers	13,166	0.93	0.88	0.95	1.02	0.96	1.03 (0.92–1.16)
9	Ischemic heart disease	13,036	1.20	0.99	0.77	0.77	0.83	0.69 (0.61–0.79)
10	Renal diseases	12,853	0.61	0.82	1.00	1.03	0.98	1.59 (1.34–1.89)
11	Lung cancer	9310	1.02	0.84	0.81	0.82	1.01	0.99 (0.85–1.14)
12	Remainder of diseases of the respiratory system	9243	0.78	0.89	1.04	1.00	0.98	1.27 (1.12–1.43)
13	Liver diseases	7824	0.75	0.82	0.96	0.93	1.10	1.46 (1.26–1.69)
14	Diabetes	6853	0.59	0.76	0.92	0.92	0.75	1.26 (0.98–1.62)
15	Remainder of diseases of the digestive system	5504	0.76	0.83	0.87	0.96	1.13	1.50 (1.30–1.73)
16	Chronic lower respiratory diseases	5410	0.80	0.88	0.73	0.77	1.02	1.29 1.06–1.56)
17	Remainder of diseases of the circulatory system	5304	0.90	0.94	0.86	0.89	1.03	1.15 (1.02–1.31)
18	Tuberculosis	4467	0.78	0.91	0.94	0.83	1.03	1.31 (1.11–1.55)
19	Drowning	3984	0.67	0.82	0.96	0.91	1.14	1.70 (1.43–2.02)
20	Remainder of diseases of the nervous system	3949	0.62	0.90	0.88	0.93	1.07	1.74 (1.45–2.09)
21	Self-harm	3761	0.46	0.80	0.78	0.96	1.12	2.44 (1.94–3.07)
22	HIV	3637	0.90	0.83	0.81	0.82	1.06	1.19 (0.99–1.42)
23	Assault	3300	0.62	0.97	0.81	0.72	0.64	1.03 (0.78–1.36)
24	Remainder of certain infectious and parasitic diseases	2667	0.80	0.90	0.88	0.93	0.92	1.16 (0.96–1.39)
25	Certain conditions originating in the perinatal period	2573	0.93	0.87	0.77	0.69	0.57	0.62 (0.48–0.79)
26	Hypertensive heart disease	2478	0.77	0.77	0.72	0.65	0.79	1.02 (0.78–1.34)
27	Colon and rectal cancer	2306	1.34	0.85	0.68	0.63	0.74	0.56 (0.45–0.69)
28	Leukemia	2024	0.95	0.91	0.91	0.84	0.90	0.94 (0.80–1.12)
29	Remainder of diseases of the genitourinary system	1851	0.94	0.78	0.89	0.80	0.90	0.96 (0.77–1.19)
30	Lip, oral cavity, and pharynx cancer	1839	1.08	0.84	0.88	0.77	0.92	0.85 (0.70–1.03)
31	Meninges, brain and other parts of central nervous system cancer	1785	0.78	0.85	0.85	1.01	0.92	1.17 (0.97–1.43)
	All causes	411,279	0.88	0.93	1.07	1.06	1.09	1.25 (1.47–1.72)

Cause of death with Q5/Q1 ratios greater than unity suggests a higher risk of death among the deprived compared to the non-deprived super-districts. Of the 31 leading causes of deaths, eight: unspecified causes, liver and bile duct cancer, traffic injuries, renal diseases, remainder of diseases of the digestive system, drowning, remainder of diseases of the nervous system, and self-harm, had Q5/Q1 ratios greater than 1.5. Perinatal disorders and colorectal cancer had Q5/Q1 ratios less than 0.66. Ischemic heart disease had higher SMR among the most affluent super-districts.

### Mortality clustering

#### Selecting the number of clusters

The elbow plot of R^2^ by number of clusters shown in Additional file 1: did not exhibit a very strong elbow. The line started to plateau after five clusters. We chose six clusters since it meant that the deep south region was a separate cluster. This is consistent with the fact that the deep south is predominated by the Malay-Muslim ethnic group, which have their own distinct health problems [4].

#### Cluster description

**Fig. 3:**
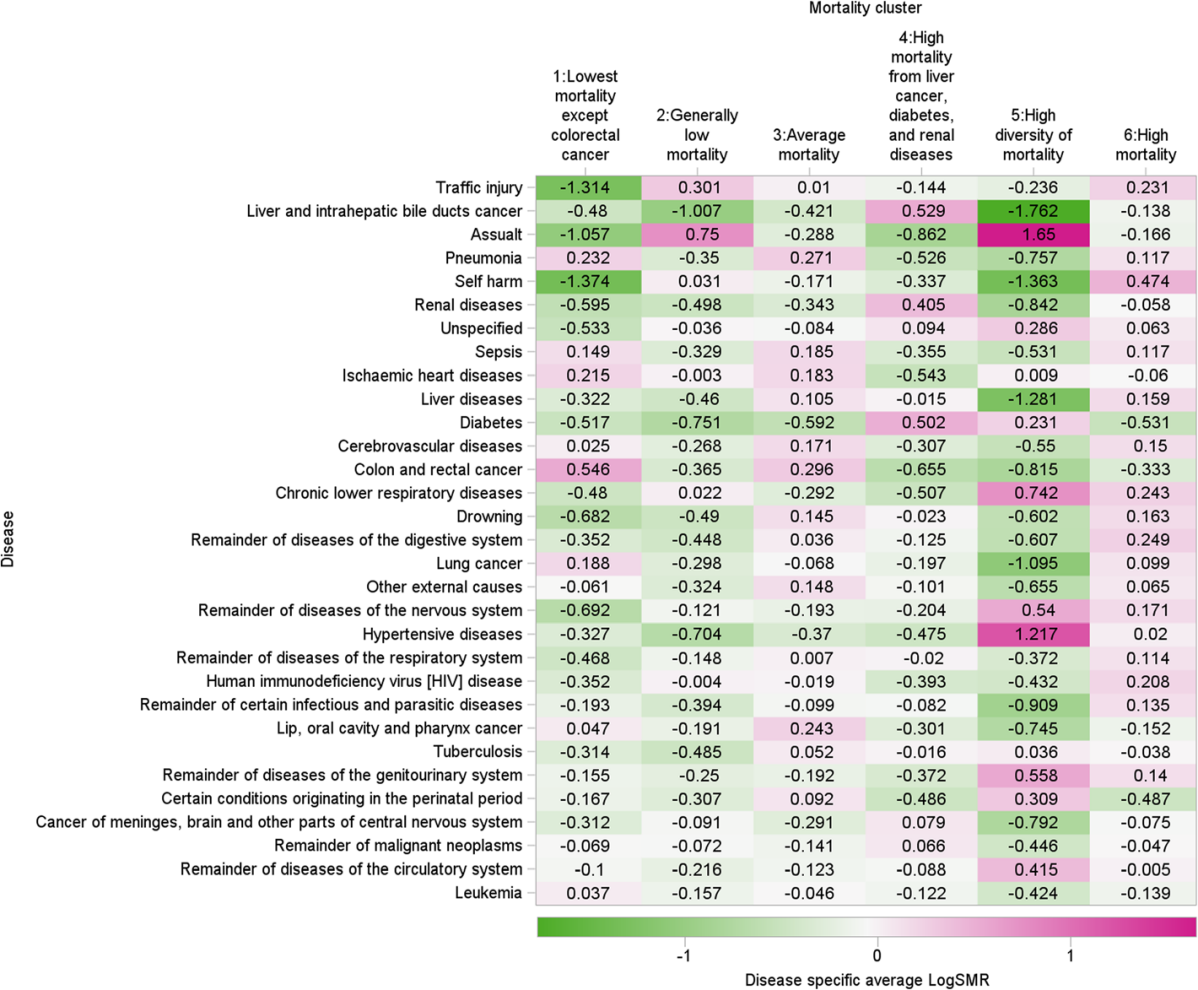
Cause-specific mortality for each mortality cluster (The color/hue of each cell indicates the level of SMR, higher value in dark pink and lower value in dark green)

Fig. 3 shows a heat map displaying logarithms of the SMR for the 31 disease groups stratified by mortality cluster. The diseases are sorted in decreasing order by their R^2^ value with the highest at the top.

Cluster 1 consists of super-districts with low mortality (green shade) for many causes. Death from traffic injury and assault were distinctly low whereas death from colon and rectal cancer were notably high.

Cluster 2 contains super-districts having low mortality rates from various causes but assault and traffic injury was exceptionally high.

Cluster 3 includes super-districts with average standardized mortality rates.

Cluster 4 consists of super-districts with high mortality from liver cancer, diabetes, and renal disease. In contrast, mortality due to ischemic heart disease in this cluster was lower than any other cluster. Cluster 5 represents super-districts with a high level of assault and hypertensive heart disease, and extremely low mortality due to self-harm, liver cancer, liver disease, and lung cancer.

Cluster 6 contains super-districts where most cause-specific mortalities were higher than the national average.

#### Interpretation from the cluster analysis

**Fig. 4:**
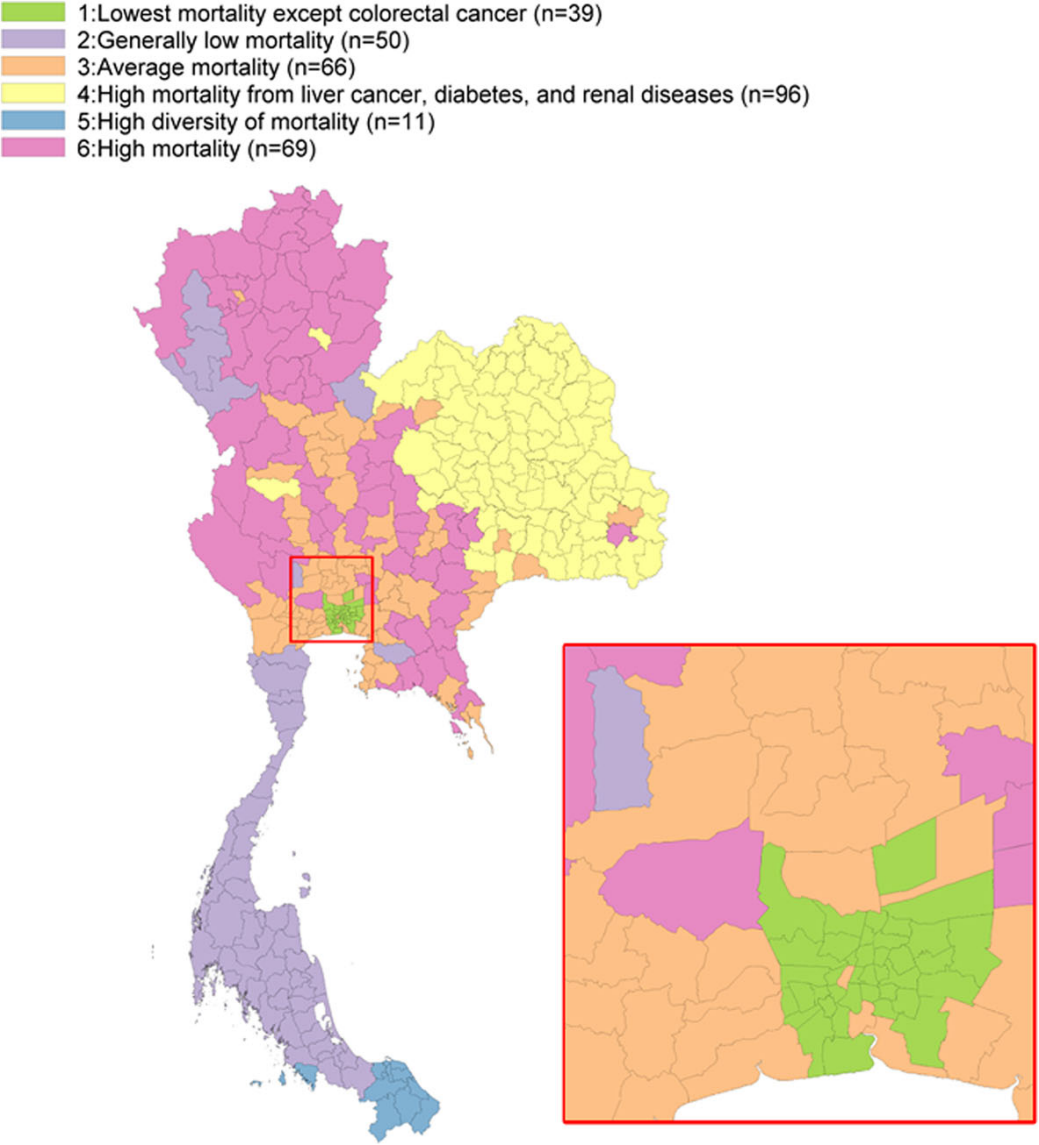
Geographical distribution of mortality clusters

These six clusters of causes of death were named as follows: cluster 1 “lowest mortality except colorectal cancer”, cluster 2 “generally low mortality”, cluster 3 “average mortality”, cluster 4 “high mortality from liver cancer, diabetes, and renal diseases”, cluster 5 “high diversity of mortality”, and cluster 6 “high mortality”. Results of this cluster analysis are depicted as a choropleth map and shown in Fig. 4 which suggests a strong geographic clustering of mortality. The location of each cluster is as follows; cluster 1: Greater Bangkok; cluster 2: southern region excluding the deep south area; cluster 3: central region; cluster 4: northeastern region; cluster 5: deep south region; cluster 6: northern, eastern and western regions.

### Socioeconomic clustering

#### Selecting the number of clusters

The elbow plot of R^2^ for socioeconomic clustering, shown in Additional file 2, had more than one distinct elbow. Possible choices for the number of clusters were 5, and 7. We chose 5 clusters as it resulted in being able to distinguish Bangkok from the rest of central Thailand, while 7 clusters resulted in some clusters having too few super-districts.

#### Cluster description

**Fig. 5:**
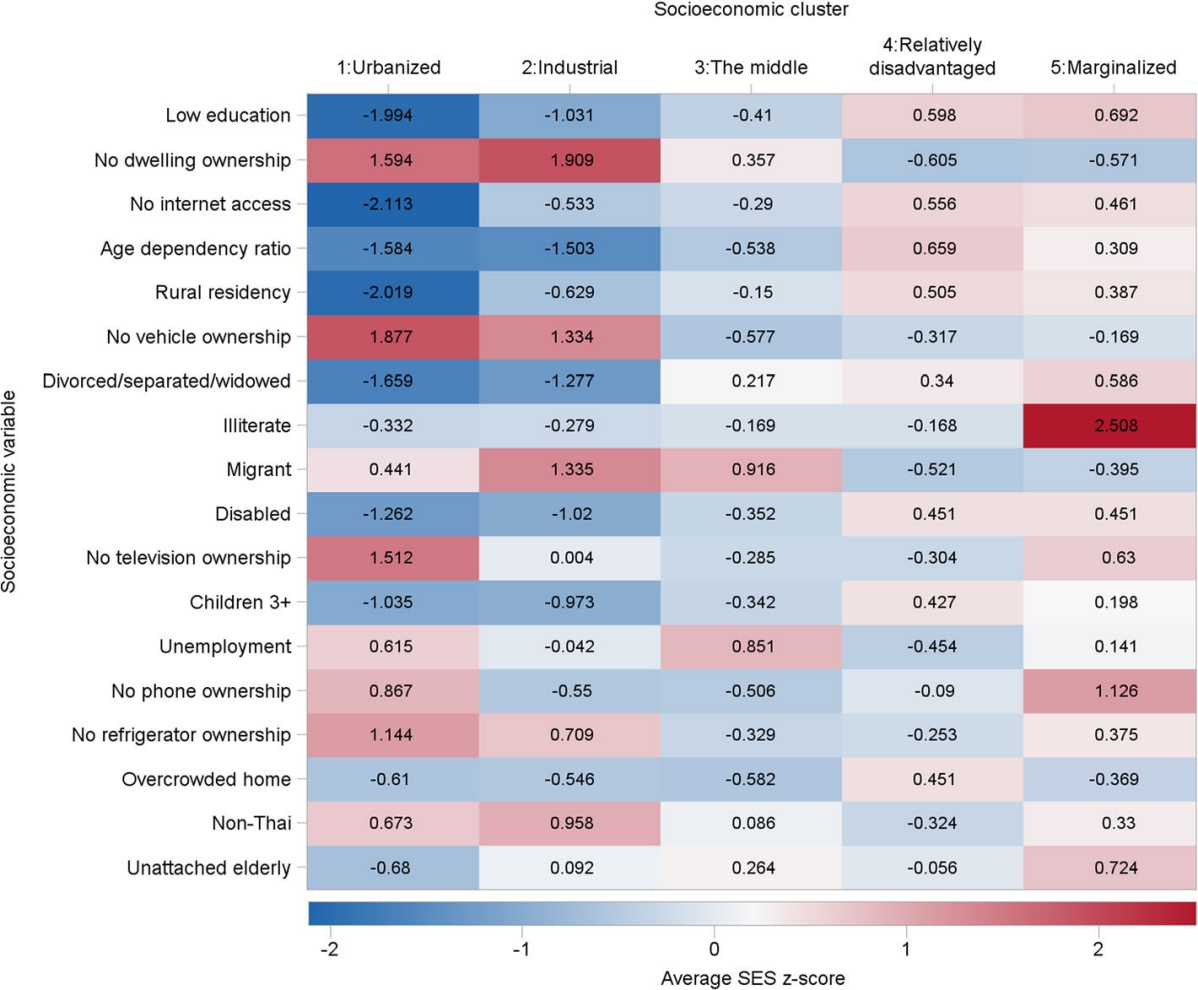
Average z-scores of socioeconomic and demographic characteristics for each socioeconomic cluster (The color/hue of each cell indicates the level of SES, higher values in dark red and lower values in dark blue)

Fig. 5 shows a heat map of the cluster analysis of socioeconomic and demographic characteristics for all super-districts. Five socioeconomic clusters emerged reflecting a gradient of socioeconomic development and urbanization.

Socioeconomic cluster 1 was highly urbanized. This cluster is represented by the well-educated, a high proportion of working aged people, low level of unattached elderly who live alone, and low level of divorced/separated/widowed females. The average household size was small, not crowded and contained a high proportion of people with access to the internet.

Socioeconomic cluster 2 is represented by those with a low overall level of socioeconomic advantages. However, the proportion of residents living in rental dwellings and the concentration of migrants and foreigners were high in this cluster. This is characteristics of industrialized or manufacturing areas.

Socioeconomic cluster 3 fell in-between all other socioeconomic types and showed a slightly higher socioeconomic advantage than the national average.

Socioeconomic cluster 4 represents the disadvantaged areas. The overall socioeconomic levels were below the national average. This socioeconomic cluster was over-represented by households located in non-municipal areas with larger members per households, overcrowded households, a high proportion of elderly and children, and having limited access to the internet.

Finally, socioeconomic cluster 5 was also characterized as a disadvantaged area with a notably high proportion of low educated and illiterate individuals.

#### Interpretation from the cluster analysis

**Fig. 6:**
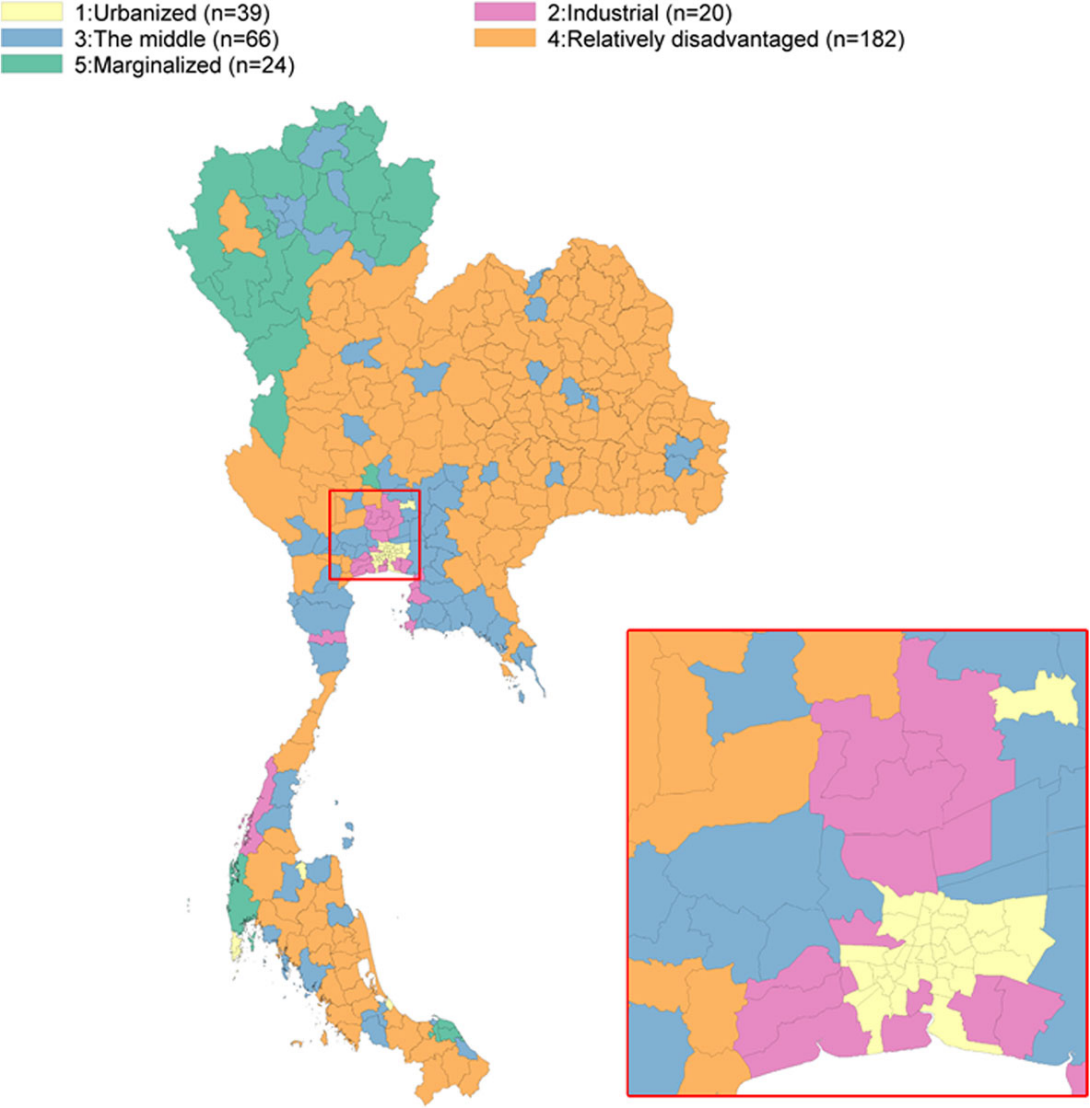
Geographical distribution of socioeconomic clusters

Based on this cluster analysis of socioeconomic profiles of super-districts, we named these five respective clusters as “urbanized”, “industrialized”, “the middle”, “relatively disadvantaged”, and “marginalized”. Fig. 6 shows a choropleth map of the geographical distribution of the socioeconomic clusters, suggesting spatial clustering. Socioeconomic cluster 1 (urbanized) contains super-districts located in Bangkok and some provincial capital cities in the southern region; cluster 2 (industrialized) contains super-districts mainly located in the vicinity of Bangkok; cluster 3 (the middle) contains super-districts located in the lower part of the central and eastern regions, and some major cities scattered throughout the country; cluster 4 (relatively disadvantaged) contains super-districts located in the central, northeastern, and southern regions; and cluster 5 (marginalized) contains super-districts mostly located at the border areas of Myanmar and Malaysia.

### Association between mortality and socioeconomic clusters

Table 3 shows a tabulation of the 331 super-districts classified by mortality cluster and socioeconomic cluster. The “urbanized” cluster had the largest proportion of good health while the “marginalized” cluster had the largest percentage of poor health and higher mortality.

**Table 3:** Distribution of super-districts by six mortality clusters and five socioeconomic clusters (*N* = 331)

Mortality cluster	Socioeconomic cluster					
	1:Urbanized	2:Industrial	3:The middle	4:Relatively disadvantaged	5:Marginalized	Total
1:Lowest mortality except colorectal cancer	32	5	2	0	0	39(12%)
2:Generally low mortality	4	2	13	28	3	50 (15%)
3:Average mortality	3	13	25	25	0	66 (20%)
4: High mortality from liver cancer, diabetes, and renal diseases	0	0	6	90	0	96 (29%)
5:High diversity of mortality	0	0	2	6	3	11 (3%)
6:High mortality	0	0	18	33	18	69 (21%)
Total	39	20	66	182	24	331
	12%	6%	20%	55%	7%	100%

*Fisher’s exact test with 10,000 Monte Carlo simulations (p-value < 0.0001)*

About 55% of all super-districts in Thailand were classified as relatively disadvantaged (182 super districts belonged to cluster 5), of which 90 (27% of total super-districts) had a higher mortality due to liver cancer, diabetes, and renal diseases. In addition, 24 super-districts (7%) were classified as marginalized, of which 8 had a high mortality rate. The association between mortality and socioeconomic patterns was highly significant (*p*-value <0.0001).

## Discussion

Based on principal components analysis, several common diseases were found to have higher excess deaths among the low socioeconomic status super-districts than the more affluent ones. The exception was colorectal cancer, which was more common among the urbanized super-districts. Cluster analysis revealed a clear clustering of cause-specific mortality and socioeconomic status characteristics by geographical area. These cause-specific mortality clusters were associated with different types of socioeconomic clusters.

Principal component analysis and cluster analysis complement each other as analytical tools and can be effectively used to identify clusters based on socioeconomic characteristics. Results from both PCA and cluster analysis on mortality concurred with prior studies which demonstrate that deprived groups have higher overall mortality [9, 32, 33]. Socioeconomic status strongly influences the availability of and access to health services, exposure to environmental hazards, and social cohesion [34]. Several studies in Thailand have also shown that lower socioeconomic groups have poorer health [35, 36], higher mortality [37, 38], a higher prevalence of smoking [39], and a higher prevalence of renal diseases [40] compared with national averages.

Our PCA and cluster analysis found higher mortality rates of colorectal cancer in the more advantaged areas. This finding concurs with previous studies in Thailand and China, which reported a higher incidence and mortality from colorectal cancers in urban areas than in rural areas [41, 42]. However, other studies reported that urban populations and those with high socioeconomic status are more likely to receive colorectal cancer screening and higher frequency of fruit and vegetable consumption, which are known to reduce the risk of colorectal cancer [43, 44]. Other risk factors may play more important roles in fatal colorectal cancer in urban populations, such as inactive lifestyle, smoking and tobacco use, overweight and obesity, and low-fiber and high-fat diet.

Although Thailand has substantially reduced poverty and achieved considerable gains in the health status of its population over recent decades, poor health attributed from poverty remain significant. Our study found that 55% of the Thai population resides in relatively disadvantaged areas, for which a high proportion have high mortality due to liver cancer, diabetes, and renal diseases. These areas are exclusively located in the northeastern region. The high mortality of liver cancer geographically coincides with endemic areas of Opisthorchiasis in the northeast [45–47]. Previous reviews reported similar findings that low socioeconomic groups were more likely to die from diabetes and renal diseases [48, 49]. Also, diabetes and hypertension are two major contributing factors to end stage renal diseases. Effective interventions, both biomedical and behavioral, are required to prevent excess deaths from liver fluke. Mortality in this study was clustered by both overall and cause specific mortality. Populations with high mortality rates were clustered in the northern and remote mountainous areas in the west where livelihoods depend heavily on agriculture as a major source of income. In contrast, areas of generally low mortality were clustered mainly in the southern region where people are better off, relying on tourism and lucrative rubber and palm plantations. The other three clusters were grouped by exceptional excess mortality for one or more specific groups. For example, the extremely higher rates for assaults of “high diversity of mortality” cluster in the deep south region reflected the armed conflict situation, where assaults and violence have occurred in the southern part of Thailand since 2004 [50].

One possible limitation of this study is that the quality of mortality statistics was considered poor because a large proportion of registered deaths are classified as being due to ill-defined conditions [51, 52]. Unspecified causes of death were also more common in the deprived area whereas perinatal problems were less common. These two phenomena may be explained by different mortality registration biases. The quality of mortality statistics varied by administrative districts in terms of the completeness of death registration and the accuracy of cause-of-death attribution [51–53]. Higher socioeconomic areas had better availability of access to health care and accurate causes of death were more likely to be clinically certified by medical personnel. In contrast, approximately 70% of deaths in rural areas occurred outside hospitals where cause of death is recorded and coded by non-medical personnel who may not be able to specify an accurate cause of death [54]. Perinatal deaths in rural areas were also more likely to be ignored or unregistered [55, 56], thus rural areas will appear to have lower perinatal death rates.

Another limitation is that selection of socioeconomic variables and specific causes of death was critical in this study. Use of different variables or causes may yield different results. We used only the first principal component to construct the deprivation index, thus our index may not have accounted for the contribution of all socioeconomic variables or explained a sufficient amount of variability [57]. The K-means algorithm is a well-known clustering algorithm due to its robustness and efficiency in analyzing large datasets; however, it requires the number of clusters to be pre-specified. Finding the appropriate number of clusters for a given data set is somewhat arbitrary and the final choice can affect the results. [58].

## Conclusion

Socially deprived areas had an excess of overall and cause specific deaths. Specific policy measures in reducing the socioeconomic gap which contribute to a reduction in health inequity can be guided by the geographical distribution of socioeconomic clusters. The affluent areas, despite low general mortality, still have many preventable deaths such as colorectal cancer. Further epidemiological studies are needed to examine certain causes of excessive deaths in the non-deprived areas.

## Additional files

Additional file 1: Elbow plot of R2 for selection of number of cluster by K-means cluster analysis of mortality data K-means cluster analysis of mortality data. (PNG 16 kb)

Additional file 2: Elbow plot of R2 for selection of number of cluster by K-means cluster analysis of socioeconomic data. (PNG 16 kb)

## Abbreviations

PCA: Principal component analysis
SMR: Standardized mortality ratio
