# Supplementary figures and images for: Area-level socioeconomic deprivation and mortality differentials in Thailand: results from principal component analysis and cluster analysis
Source: Int J Equity Health. 2017 Jul 3;16:117. doi: 10.1186/s12939-017-0613-z (PMC5496369; doi:10.1186/s12939-017-0613-z)

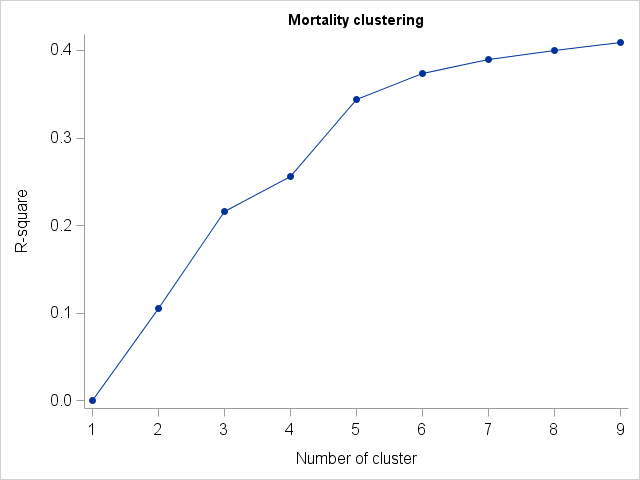

Supplement: Supplementary file 1 — Elbow plot of R2 for selection of number of cluster by K-means cluster analysis of mortality data K-means cluster analysis of mortality data. (PNG 16 kb) [file 12939_2017_613_MOESM1_ESM.png]

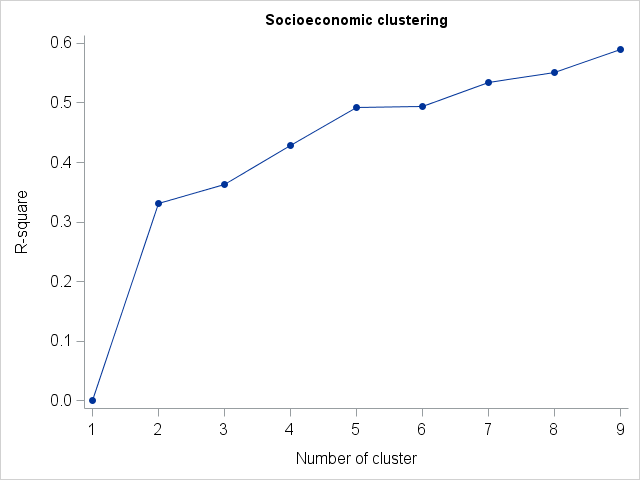

Supplement: Supplementary file 2 — Elbow plot of R2 for selection of number of cluster by K-means cluster analysis of socioeconomic data. (PNG 16 kb) [file 12939_2017_613_MOESM2_ESM.png]
